# The effect of competition on discrimination in online markets—Anonymity and selection

**DOI:** 10.1371/journal.pone.0221857

**Published:** 2019-08-28

**Authors:** Emma von Essen, Jonas Karlsson

**Affiliations:** 1 Swedish Institute for Social Research, Stockholm University, Stockholm, Sweden; 2 Department of Economics and Business, Aarhus University, Aarhus, Denmark; 3 Independent Researcher, Stockholm Internet Research Group, Stockhlm, Sweden; Universitat Jaume I, SPAIN

## Abstract

Empirical studies show that discrimination by identity found in offline markets also prevails online. This paper reveal that in a competitive market, buyers that intend to discriminate exist but they are prevented from influencing the market outcome. To this end, we construct a field experiment on eBay, where half of the sellers disclose their names in their usernames while the other half do not. eBay, however, automatically discloses the seller’s names to the buyer after the auction. In the anonymous auctions, winning bidders thus learn the identity of the seller after the auction ends, and here we find buyers to discriminate against sellers with foreign-sounding names by leaving them feedback less often. However, there is no such discrimination in feedback provision when the seller name was known to the buyer before the auction. When bidders know the names of the sellers, the bidders with animus towards individuals with specific names can select out of auctions from these sellers, leaving winners that do not discriminate. One would expect that the auctions of for example sellers with foreign-sounding names would receive fewer bidders and thus lower auction prices. However, we observe no such differences: there are no statistically significant differences in the number of bids or auction prices received by sellers with foreign or domestic sounding names.

## Introduction

There is an extensive social science literature showing that women and minority groups end up with worse economic outcomes in offline markets compared to other groups, [1, 2]. Today much buying and selling occur online, and the discrepancies in economic outcomes are also found here. For example, minority groups receive lower sale prices compared to other groups [3–6]. On the internet, trust cannot be generated through inspecting the sellers’ clothes, looking them in the eyes, shaking their hands, or physically inspecting the product before buying. Here, reputational feedback systems generate trust and thereby sustain economic exchange online, e.g. [7–10]. This paper shows that in a competitive market, buyers that intend to discriminate are present, but they cannot influence the market outcome.

A tasted-based explanation of discrimination in the sale price or feedback rating assumes a dislike (or like) of a particular group, leading to a selection of whom one buys from [11, 12]. An explanation by statistical discrimination assumes that buyers believe certain sellers to have a worse delivery or feedback behavior (assuming the same quality of good) [13, 14]. If there are enough non-discriminatory bidders, taste-based discrimination should not play a role. However, theoretically, a general social custom can sustain discrimination in small markets, [15], with random trading, even if there is a significant minority of non-discriminatory buyers. Because there will be no alternative trading partner at the time of trading. If discriminatory bidders avoid specific sellers (selection), these sellers will have fewer bidders, which lead to a lower sale price. An increase in the number of bidders should decrease the impact of the marginal discriminatory bidder, [11]. A market with many buyers and sellers will have enough bidders for all sellers, and the market price might not be affected. Some previous empirical literature shows that discrimination in the sale price is smaller, or not present, in competitive markets [3, 4, 6, 16]. These studies have, however, not yet shown that the discriminatory bidders are prevented from influencing the market outcome, such as the sale price. Buyers with an animus might, for example, not be present in these markets. We believe that we have found a way to show this.

To this end, we set up an online field experiment on Swedish eBay, selling a homogeneous product and observing buyer behavior. Swedish eBay is a consumer-to-consumer platform, where buying and selling involves a sealed-bid second-price auction. While bidding, the information known between bidder and seller is their usernames and previous feedback. After the auction is completed, but before feedback can be provided, eBay automatically reveals the real names of the seller and buyer (winning bidder). We collaborate with real persons creating new seller accounts on eBay. Their names signal their gender and foreignness, from a stereotypical Swedish perspective—generating four different identity groups. We asked half of the sellers to create usernames that disclose their real names in their usernames, and the other half use anonymous usernames. We then manage all seller accounts and use identical conduct in all auctions. When the seller name is visible bidders with an animus towards individuals of specific groups can select auction based on seller username, thus avoiding sellers of their dislike. When name is not visible, we block the possibility for buyers to avoid sellers of their dislike by using anonymous usernames. By investigating discrimination in the provision of feedback, we detect whether there are bidders with animus in the market. Here, we expect discrimination by gender and foreignness in buyer feedback. Bidders willing to discriminate might avoid auctions from specific sellers when the name is visible. Auctions of sellers with, for example, foreign-sounding names could thus receive fewer bidders and lower auction prices. However, since we consider the voucher market to be competitive, we expect no or little discrimination in the sale price, e.g. [3, 6].

We find discrimination in buyer feedback provision of foreign male sellers. Importantly, the discrimination is only present among the buyers that bid for anonymous sellers and could not select seller on username (social identity). Only bidders that win the auction can provide feedback to the seller; thus, when bidders avoid bidding on items from specific sellers, it also implies that they cannot provide feedback to these sellers. There is no buyer feedback discrimination in the group where the seller names were visible during the bidding process. Buyers with an animus seem to avoid sellers of their dislike if they have the identity information. This buyer behavior could potentially lead to fewer bidders among the sellers with, for example, foreign-sounding names and a lower sale price. However, we find no discrimination in the sale price. In our market, the number of bids is also similar across seller groups. The results suggest that discriminatory buyers do not seem to affect the sale price directly in a competitive market.

Unfortunately, we do not have the data to explicitly test whether the lower feedback rates brings about a lower probability of sales in later auctions. Across our experiment, all vouchers for sale were sold. Following previous literature, the type of discrimination we detect might lead to fewer sales and lower prices in future auctions. [7, 8, 17–23].

The paper is structured as follows. The coming section describes and related literature. Next, we describe our design, hypotheses before we discuss the data and results. Last, we discuss limitations and conclude.

## Related literature

Our paper relates primarily to three strands of literature. First, we discuss the literature investigating discrimination by gender and foreignness in online buying and selling situations. Second, the behavioral economics literature on discrimination by gender and foreignness in trust and reciprocity is relevant for our study. Third, we discuss the labor economics literature on the anonymous application procedure.

In experimental discrimination research, names are a common way of studying the effects of gender or race, e.g. [24, 25]. Closest to our study is a paper using distinct black and white names in the usernames to detect discrimination in sale prices on eBay, [3]. An issue regarding the use of names when studying discrimination is the possibility that they signal more than gender and foreignness, such as social and economic class or geographic region. The seller names we have chosen are common in various regions and social classes [26]. Online, names are the most common cues that individuals manipulate, conceal, or reveal. Using names in our study allows us to investigate the intersection of gender and foreignness in a relatively controlled way. Focusing on only one social dimension can disguise possible discrimination. Previous research on race and gender analyzed separately can lead us to believe that women with foreign names face double discrimination. However, when the two dimensions are analyzed together, previous research finds males with foreign names to fare worst in a labor market context, e.g. [27].

A trust game can well describe buying and selling online, [18, 28]. When buying the good, the buyer needs to trust the seller to return the good. The feedback system generates reputation of the sellers’ (buyers’) previous sales, making them trustworthy or not. Considering that most buyers and sellers interact only once, there are, however, no direct economic incentives to provide feedback, but feedback is nevertheless observed to a large degree. Trust and reciprocal behavior in other contexts seem to depend on social identities where, if anything, women and minorities are disfavored, e.g. [29]. Feedback ratings seem to foster trust through reputational and retaliation effects and by agents avoiding negative feedback [30, 31], and these effects tend to lead to feedback being mostly positive, e.g. [32], and leaving no feedback is in previous literature seen as a sign of discontent or avoidance, e.g. [33].

The reputation feedback is thus relying on indirect reciprocity, i.e., a cyclical network (see for example [34]). Feedback follows downstream indirect reciprocity, where A was nice to B, and therefore C is nice to A. In most auctions, the buyer provides the feedback before the seller [32]. Many previous studies explain the provision of feedback by reciprocal preferences [17, 29, 35–37]. From an economic theory perspective, introducing other-regarding preferences, such as reciprocity, will not change the equilibrium strategies in a second price auction, if the bidders have incomplete information [38]. Empirically, a few laboratory experiments explore discrimination in trust and reciprocity by gender and race, see a review by [29]. If anything, studies have found evidence of discrimination in trust to the disadvantage of minority groups, e.g. [39–44]. In a similar vein, some studies find reciprocity to differ by recipient [40, 42, 44], while other studies find no such differences [39, 41].

Anonymity is a contentious issue, and a common part of online behavior, e.g. [45]. Individuals can to a greater extent compared to offline choose not to convey social information (be anonymous) in their usernames or emails to, for example, avoid discrimination [46]. Anonymity can create a fair evaluation (see [47] for a brief review). In their seminal paper [48], the author reveals discrimination of female musicians in hiring, by using anonymous auditions—where applicants perform behind a curtain. Another paper shows that if job applicants do not disclose their names, women can increase their probability of being contacted by an employer [49]. Closer to our setting are two papers, [50] and [51], which show that in Sweden and Holland an anonymous application procedure increases the probability of being interviewed (initial stage), for women and individuals with foreign names, as well as increased women’s chances of receiving a job offer (after anonymity is lifted).

## Design of the study

The study is conducted on eBay Sweden selling cinema vouchers. Swedish eBay requires a social security number connected to each account; it is thus not possible to create a new account to clean one’s reputation. We collaborated with a group of individuals who created new eBay accounts using their name and social security number. To provide payment information, we created Hotmail accounts using their name. We then initiated auctions and recorded buyer behavior.

To select the groups of collaborating sellers, we let an external group of students at Stockholm university categorize names of potential sellers we could collaborate with, by gender and foreignness. We then chose the names of the sellers that the students best could classify (from a stereotypical Swedish perspective; please see Procedure in S1 Appendix for a description of the procedure). The sellers have no previous record of buying or selling on eBay. Half of our sellers created usernames consisting of their real first names (non-anonymous), while the other half created usernames consisting of their initials and a two-digit number (anonymous). In total, we have eight different treatment groups (see Table 1 below). In all, we have sixteen sellers, thus two sellers in each of the eight groups. The bidders are unaware of being part in an experiment, and will therefore not be able to adjust their behavior to any experimenter expectations. Our sellers are also passive without the ability to discriminate against buyers.

**Table 1:** Seller name groups*.

Non-Anonymous usernames	Anonymous usernames
Foreign male names	Foreign male names
Non-foreign male names	Non-foreign male names
Foreign female names	Foreign male female names
Non-foreign female names	Non-foreign female names

*The list of actual seller names can be found in Table A in S1 Apendix.

The part of our design where sellers display their real names in their usernames is similar to previous studies of online sale price discrimination, and here the buyers can select into the auction based on seller name. The design novelty of our study is the part where sellers use anonymous usernames. This part mimics a randomized controlled experiment. The two parts will, therefore, be analyzed separately.

We randomly chose four sellers, two anonymous and two non-anonymous, to place a pair of cinema vouchers on eBay. Two were randomly picked to start before lunch and the other two after lunch. We used five days as the length of the auctions, as this seemed to be the average length of an auction in the voucher market. The design implies that two of our anonymous seller groups and two of our non-anonymous seller groups always sell simultaneously. The design also implies that all our seller groups overlap in their auctions and compete for bidders. In the analyses, we control for the start date of the auction and control for the number of bids in our analyses. Table 2 displays the timing of events of the study.

**Table 2:** The timing of events.

Events
*Username and feedback of the seller is visible to the buyer*.
1. The seller places the pair of vouchers on eBay.
2. The bidding starts.
3. The auction closes.
*The real name of seller visible to the buyer*.
4. eBay sends an email to the buyer and the seller.
5. The seller sends an email with payment information.
6. The buyer pays for the vouchers.
7. The seller ships the vouchers
8. The buyer and the sellers can provide feedback.

To keep the auctions and sellers similar across groups, we used the same seller conduct acting as ‘perfect sellers’, avoiding negative feedback from the buyer. We shipped the vouchers such that all buyers received them within the same time frame. eBay also conveys addresses of buyers and sellers. Identity can sometimes be revealed through other information present in online trade, such as location [52]. To keep everything constant, apart from names, across seller groups, we exogenously impose different street names from the same local residential area from Stockholm. We control for the imposed street names in the regressions. We also randomized similar standardized texts and standard pictures taken from SF Studios (the largest film distributor in Sweden). The lowest bid was set to 1 SEK in all auctions.

Bidders submit their maximum bids (willingness to pay) and eBay bid for the users by proxy. As more buyers submitted their bids, it rises by the minimum increment until the second-highest bid is surpassed. The auction framework should provide a setting to study discrimination in buyers’ willingness-to-pay. However, this structure has been shown to display reactive bids, such as last-minute bids [53–55]. According to a simple model, bidders in a market like eBay, with many traders, will explore trading opportunities with all sellers before raising the bid [56]. Assuming the share of bidders with an animus against certain sellers are the same for reactive and non-reactive bidders. Then we would not expect the reactive bidders to affect the sale price across the groups of sellers. Reactive bidders seem to be less experienced in buying and selling [54], and in our analyses, we control for buyer experience.

All sellers started with no previous feedback. The number of feedback ratings became endogenous during the experiment. Before a buyer or a seller has received ten unique instances of feedback, the account page contains a symbol indicating that the seller or buyer is new to eBay. In the analysis, we control for the day of auction and whether the sellers are labeled as new to eBay or not. We wait for the buyer to provide feedback to our seller. Previous research found that in 85% of the cases on eBay buyers rate sellers first [32]. After a buyer rates us, we give standardized positive feedback to the buyer. To capture buyers that only provide feedback after the seller does, we give feedback to any remaining buyer after ten days, muting possible buyer beliefs of sellers not returning feedback. The threshold of ten days is based on the findings of [57]. eBay immediately displays the feedback rating on the individual’s public account page.

The measures we collect are price, provision of feedback and buyer characteristics. The buyers’ names we categorize in the same way as the sellers’. We also note the date the auction ended and the date the buyer provided feedback. The experiment ran across four months, which allowed us to conduct approximately 55 auctions in each named group (436 auctions in total). Table 3 presents the sample size across the seller identity groups.

**Table 3:** Sample size (number of buyers) across the treatment groups.

Treatment group	n
*Non-anonymous seller group*	
Non-foreign male	56
Foreign male	51
Non-foreign female	53
Foreign female	58
*Anonymous seller group*	
Non-foreign male	54
Foreign male	55
Non-foreign female	55
Foreign female	54

### Competition in the market

The market for vouchers on eBay seems to contain a reasonable number of sellers and buyers. Cinema vouchers are a common and homogeneous good for which the price and quality are common knowledge. People buy and sell vouchers offline as well as online and frequently give them as presents. Cinema vouchers can be bought at most cinema venues anywhere in Sweden as well as online at Swedish cinema (www.sf.se) to a fixed price. There is also a service at Swedish Cinema where the customer can double-check that the voucher is valid. Today Swedish cinema visits amount to 16.5 million per year; thus around 1.3 million cinema visits per month.

In retrospect, we wish that we had collected information regarding the number of auctions at the time of the experiment. In previous research, the number of competing sellers on online advertisement websites is used as a proxy for competition [3, 6]. In markets with more than 20 ads per week, discrimination is no longer present in the sale price, and these markets are considered competitive. Today, there are about 40-50 auctions selling cinema vouchers on Swedish eBay every day, with about five days as the average length. The market for cinema has not changed, there is no reason to believe that the number of auctions was lower during the experiment.

We did not collect the number of unique bidders per auctions, which would have provided us with a proxy of the potential number of bidders. We are, however, grateful to an anonymous referee that pushed us to go through screen dumps we had saved from most of the sales we conducted (341 out of 436). The dumps cannot be connected to a specific auction but contain seller username and the bidders. The average number of unique bidders in the market during the experiment was 7.8., and there are no significant differences across seller groups, or across anonymous vs non-anonymous sellers (see Table R in S1 Appendix for full results). A previous paper selling baseball cards on eBay [5] have a bid amount of 3.353 (from the constant from column D of Table 3). For a non-African-American player, the average bid amount was 4.59. Assuming their bid amount is the same as their number of unique bidders, the numbers are still lower compared to the average number of unique bidders per auction in our market. In the unique number of bidders, our market seems more competitive than theirs. In sum, we consider the market for cinema vouchers at Swedish eBay to be competitive.

#### Hypotheses

After the auction ends, eBay automatically reveals the names of the winning buyer and seller to settle payment and shipping, after which they can provide feedback to each other. The reputational feedback mechanism can be viewed as well defined games [35], in which each an agent must decide if and when to provide feedback and what type of feedback to provide. There is, however, little formalized theoretical work explaining the behavior of feedback provision.

Our prior was that there are buyers with animus in the market. Within the setting when bidders cannot see the name of the seller, they will not be able to select the seller by username and this can render in a pattern of differences across seller groups in how buyers provide feedback by seller gender and foreignness. However, the bidders that faced a seller with the name in the username had full information when selecting into the bidding process. We thus expect buyers with animus not to be present in this situation due to selection. In our setting, there are four seller name groups: foreign males, foreign females, non-foreign males, non-foreign females.

*H1*: *Among the transitory anonymous sellers*, *there will be discrimination in feedback*. *We expect no feedback discrimination among non-anonymous sellers*.

In the auction, the seller’s username can affect the bids and the price. If specific sellers receive fewer bidders, this can lead to lower auction prices for these sellers. There are, however, many sellers and buyers in the market for cinema vouchers on eBay. As discussed in the previous literature section competition may lessen discrimination in the sale price.

*H2*: *Among the transitory anonymous sellers*, *there will be no differences in the average sale price*. *Among the non-anonymous sellers*, *we expect little or no differences in the average sale price*.

The previous empirical literature on discrimination in the Swedish labor market context finds the intersection of foreign and male names to sometimes fare worse than other groups [27]. According to human and computer interaction research as well as media research, the default and expected identity on the Internet is white and male (see for example [58]). We, therefore, expect discriminatory buyers to have an animus against foreign male sellers.

#### Ethical considerations

We collected verbal informed consent of all collaborating sellers (all adults). Informing them about every step of the procedure in the experiment as well as the purpose of the study. The committee vetting the ethics of research in the area of Stockholm, Sweden, approved the study design (EPN Stockholm, Nr: 2011/1328-31). Throughout the analyses, we use a significance level of 0.05. We did not take the issue of multiple testing into account when calculating the sample size. Post-hoc adjustment of the significance level would not seem to be an issue for the results of our study, see the section on Limitations and robustness.

## Data

The study ran between April 30th and August 30th, 2012, and we completed 436 full auctions. Table 4 shows the variables we collected during the experiment.

**Table 4:** Variable definitions.

Variable name	Definition
Sale price	Price paid by buyer (SEK)
Bids*	Number of bids in the auction
Feedback	1 if the buyer gave feedback, 0 otherwise
New seller	1 if the seller has <10 # of feedback, 0 otherwise
*Buyer variables*	
Buyer Female	1 if the buyer had a female name, 0 otherwise
Buyer Foreign	1 if the buyer had a foreign name, 0 otherwise
Buyer Big city**	1 if the buyer lived in a big city, 0 otherwise
Buyer New	1 if the buyer’s own feedback ≤ 10, 0 otherwise
Buyer Neg. feedback	1 if the buyer had negative feedback, 0 otherwise
Buyer Feedback	# of feedback at time of the auction
Buyer Anonymous	1 if the buyer has an anonymous username, 0 otherwise

*Unfortunately we did not collect the number of unique bidders for each auction.

**Statistics Sweden defines a big city as having more than 100.000 inhabitants.

The average price of two vouchers was 140 SEK (140 SEK, approximately 16 USD), approximately 70% of the original price. In 71% of our sales, the seller received feedback, and all were positive. The buyers in our sample are heterogeneous concerning gender, foreignness, residential area, and previous feedback. Table 5 presents descriptive statistics of the variables we have collected. Regressions are clustered on buyer username since the number of clusters is higher compared to sellers (176 vs 8), the clustering does not make a qualitative difference to the results. In 126 out of 436 auctions we as sellers provided feedback before the buyer (waiting ten days), in two-thirds of these cases the buyer never returned the feedback. The data is available via the Swedish National Data Service (https://snd.gu.se/en), reference number snd1061. We have anonymized the usernames of the buyers in the data to avoid possible identification.

**Table 5:** Descriptive statistics depending on anonymity.

	(1)		(2)		(3)	
	Total		Non-Anonymous		Anonymous	
	mean	sd	mean	sd	mean	sd
Sale price	139.828	10.674	139.445	11.081	140.211	10.262
Bids	27.683	14.625	28.922	14.947	26.445	14.223
Feedback	0.711	0.454	0.706	0.456	0.716	0.452
New seller	0.665	0.472	0.651	0.478	0.679	0.468
*Buyer variables*						
Buyer Female	0.505	0.501	0.537	0.500	0.472	0.500
Buyer Foreign	0.333	0.472	0.326	0.470	0.339	0.475
Buyer Big city*	0.227	0.419	0.234	0.424	0.220	0.415
Buyer New	0.284	0.452	0.266	0.443	0.303	0.461
Buyer negative feedback	0.310	0.463	0.339	0.475	0.280	0.450
Buyer feedback	158.131	355.240	171.408	376.289	144.853	333.205
Buyer Anonymous	0.541	0.499	0.596	0.492	0.486	0.501
Observations	436		218		218	

* Big city is defined as a city populated by more than 100.000 inhabitants (definition by Statistics Sweden).

## Results

In our sample, there are no observable differences in buyer characteristics between buyers choosing sellers with anonymous or with non-anonymous usernames (see Table H in S1 Appendix). All regressions include dummies for each seller group and are presented with and without additional control variables. We use the group of sellers with foreign male names as the reference group throughout the analysis [27]. We use interaction terms between the setting (anonymity or non-anonymity) and each of the seller groups to capture the differences in outcomes between male foreign sellers and the other groups between the anonymous and the non-anonymous setting. The control variables include whether or not the seller was new to eBay, the number of bids and the start date of the auction. Characteristics of the buyers could be considered bad controls and provide misleading results. Including the buyer characteristics in the regressions does not change the results. The Table B and C in S1 Appendix displays the results separately for the anonymous and non-anonymous situation, including buyer characteristics.

### Discrimination in the share of buyer feedback

To asses, whether there is discrimination in feedback and test whether the discrimination is larger in of the two settings: anonymous or non-anonymous, we look at the interaction terms in the regressions. Table 6 shows the marginal effects of logit regressions using the provision of feedback as an outcome variable. Regressions 1 and 2 confirm that the feedback on average does not differ between the anonymous setting and the non-anonymous setting. The three coefficients for the seller groups reveal that there are no differences in the share of provided feedback between male foreign sellers and any of the other groups when the winning buyers could view the name in the usernames of the sellers. The interaction terms, however, display that there is a pattern of discrimination in provision of feedback between seller groups in the treatment where the buyers could not see the seller names while bidding. Anonymous sellers with foreign male names receive less feedback than non-foreign females and foreign females but receive a similar share of feedback as non-foreign males. The differences in male foreign sellers and female foreign and non-foreign sellers is larger in the anonymous setting compared to the non-anonymous setting. The contrast between foreign male sellers and non-foreign as well as female foreign sellers is 18 percentage points higher in the anonymous setting compared to the non-anonymous setting (where no such differences can be detected). We also changed the reference group to female foreign sellers to probe into further contrasts. Here, we see female foreign sellers receiving less feedback compared to non-foreign females, but not the other groups (see Table E). The results are in line with the second hypotheses (H1).

**Table 6:** Logit marginal effects: Differences in share of feedback between male-foreign seller and other groups.

	(1)	(2)
	No controls	Controls
Anonymity	-0.118	-0.109
	(0.082)	(0.083)
Male non-foreign seller	-0.068	0.114
	(0.092)	(0.131)
Female non-foreign seller	-0.049	-0.047
	(0.093)	(0.092)
Female foreign seller	-0.040	-0.045
	(0.088)	(0.088)
Anonymity x Male non-foreign seller	0.154	0.149
	(0.083)	(0.084)
Anonymity x Female non-foreign seller	0.187*	0.179*
	(0.082)	(0.086)
Anonymity x Female foreign seller	0.105	0.177*
	(0.097)	(0.086)
Price		-0.002
		(0.003)
Number of bids		0.000
		(0.001)
New seller		-0.079
		(0.061)
Start-day and street name §	No	Yes
Observations	436	436

^§^ We randomized street names from the same local area across the sellers.

Standard errors in parentheses are clustered on buyer username.

* *p* < 0.05,

** *p* < 0.01,

*** *p* < 0.001

Table 6 show that we have buyers with animus in the market, avoiding to leave feedback to specific sellers. The discrimination in feedback could be due to buyers’ beliefs about the sellers’ willingness to reciprocate feedback. In the cases where our sellers provided feedback before the buyer, only in one-third of these cases buyers then provide feedback. Dropping these buyers do not change the results, see Table F in S1 Appendix. The results suggest that discriminatory buyers select sellers by names when available, but are unable to influence the market outcome.

The randomized experiment of the anonymous setting allows us to base our statistical inference on randomization tests, using sharp null hypotheses that none of the participants was affected by the treatments, following [59]. The results show that it is only among the anonymous seller that we detect differences between seller groups, the effect is primarily the differences between female non-foreign sellers and foreign sellers, see Table M and N in S1 Appendix. In previous literature, e.g. [32, 36], provision of buyer feedback lies between 65-80%. In our study, the average provision of feedback is 71%, and foreign male buyers receive feedback in on average 61% of the auctions. All other seller groups receive a larger share than foreign male buyers but within the range of what has been found in previous literature. Although our study does not have a clear baseline, the figures mentioned above lend some support to negative discrimination. Previous research suggests that buyers tend to provide more feedback to inexperienced sellers than experienced sellers [32, 33], whereas others do not confirm this [36]. We do not find indications that feedback begets feedback. In our study, the sellers all start as new sellers, and feedback accumulates across the experiment. The label as the new seller, does not seem to affect buyers provision of feedback. We have too few observations to compare the provided feedback by buyers between seller name groups at each single feedback level or comparing 0 with positive feedback. Previous research claims that five instances of feedback are enough to be treated as experienced on eBay [32]. Changing the threshold to 5 instances of feedback does not change our results qualitatively (see Table G in S1 Appendix).

Positive feedback seems to affect the probability of selling as well as the sale price [7, 8, 17–23]. All our auctions sold the product within five days, we are thus limited in saying anything on the sellers’ probability of selling the good. Conditional on receiving feedback, there is a similar pattern of discrimination in time it takes to receive feedback from the buyer. It takes an anonymous male foreign seller approximately 3-5 days longer to receive feedback compared to the other groups, see Table Q in S1 Appendix.

Our experiment cannot capture the long run effects of feedback or the effect of having zero feedback on future sale prices. The economic effect of accumulated positive feedback might be non-linear. The first few instances of feedback seem the most economically important [22], and the cumulative aspect of reputation systems can produce further inequalities [60, 61].

The discriminatory behavior we find among buyers may vary with buyer identity. It would be interesting to understand which of the four buyer groups display discrimination between the respective seller groups. In discussion with an anonymous referee, we did not include the variables on buyer characteristics in the regressions, since they may be considered bad controls and increase the omitted variable problem. Adding them does, however, not change our results. We also have too few observations in each combination of seller and buyer group to conduct a sound statistical analysis.

### Discrimination in the sale price

In the market for cinema vouchers, we find discriminatory buyers. The discrimination is only found in the anonymous setting where bidders cannot select. If discriminatory buyers avoid specific sellers when the seller names are known, this could lead to fewer bidders and lower sale price.

In Table 7 sale price is the outcome variable. We find no discriminating in sale price in any of the settings, anonymous or non-anonymous. These results are in line with the second hypothesis (H2). We conducted randomization tests for the effects of the seller groups in the anonymous setting, [59]. The results are similar and display no significant effects. See Table P and O in S1 Appendix.

**Table 7:** OLS: Discrimination in sale price between male-foreign seller and other groups.

	(1)	(2)
	No controls	Controls
Anonymity	1.194	2.437
	(2.024)	(1.717)
Male non-foreign seller	2.113	-0.016
	(2.081)	(2.984)
Female non-foreign seller	0.950	0.880
	(2.048)	(1.648)
Female foreign seller	1.270	0.978
	(1.983)	(1.585)
Anonymity x Male non-foreign seller	-0.066	-1.470
	(2.837)	(2.419)
Anonymity x Female non-foreign seller	0.323	-1.432
	(3.084)	(2.504)
Anonymity x Female foreign seller	-1.853	-3.790
	(2.797)	(2.730)
New seller		-12.613***
		(0.925)
Number of bids		-0.002
		(0.030)
Constant	138.333***	146.151***
	(1.562)	(1.786)
Start-day and street name §	No	Yes
Observations	436	436

^§^ We randomized street names from the same local area across the sellers.

Standard errors in parentheses are clustered on buyer username.

* *p* < 0.05,

** *p* < 0.01,

*** *p* < 0.001

The coefficient of the control variable “new seller”—indicating the seller’s lack of experience of buying and selling on eBay. New seller equals one if the seller has less than ten instances of feedback, and 0 otherwise. In line with previous literature, we find a lack of experience to have a negative effect on price. A new seller receives SEK 13 less than an experienced seller (*p* < 0.01) in our sample. This result implies that sellers that systematically receive fewer instances of feedback for the same number of completed auctions will need longer to reach the point where eBay removes the label of being new, and this brings them an economic loss. We, therefore, ran a regression for the non-anonymous setting separately for experienced and new sellers. Table 8 illustrate that there are no differences in price among the new sellers or the experienced sellers. However, samples sizes are small. We also tried interacting the variable New Seller and the respective identity groups of the seller, and we get similar results, Table D in S1 Appendix.

**Table 8:** OLS: Differences in the sale price comparing new and experiences non-anonymous sellers.

	(1)	(2)
	Non-anonymous new sellers	Non-anonymous experienced sellers
Male non-foreign seller	1.915	3.953
	(2.013)	(2.815)
Female non-foreign seller	0.575	-0.942
	(2.168)	(2.562)
Female foreign seller	0.209	0.263
	(2.216)	(2.416)
Constant	132.177***	152.664***
	(3.273)	(3.500)
Controls	Yes	Yes
Adjusted *R*^2^	-0.027	0.063
Observations	142	76

Standard errors in parentheses are clustered on buyer username.

* *p* < 0.05,

** *p* < 0.01,

*** *p* < 0.001

#### Differences in the number of bids

In a less competitive market, discriminatory bidders that avoid sellers of their dislike could affect the market outcome if discriminatory buyers avoid specific sellers and fewer bidders should lead to a lower sale price. In a competitive market, however, the number of bidders will be similar across groups. In our market, the number of bids is similar across seller groups. Table 9 shows the number of bids across the seller groups that use their names in the usernames. The table shows that there seem to be no systematic differences in the number of bids the sellers receive.

**Table 9:** OLS: Differences in the number of bids for non-anonymous sellers.

	(1)	(2)
	No controls	Controls
Male non-foreign seller	1.783	1.421
	(2.670)	(2.586)
Female non-foreign seller	1.995	2.060
	(2.841)	(2.781)
Female foreign seller	1.321	1.290
	(2.445)	(2.346)
New seller		7.546***
		(2.098)
Constant	27.627***	25.162***
	(1.857)	(3.394)
Start-day and street name	No	Yes
Adjusted *R*^2^	-0.011	0.047
Observations	218	218

Standard errors in parentheses are clustered on buyer username.

* *p* < 0.05,

** *p* < 0.01,

*** *p* < 0.001

In sum, we find buyers with an animus against foreign male sellers, but in a competitive market, they are not able to influence the market outcome—the sale price. The anonymity of sellers during the bidding process prevents a bidder from selecting sellers by social identity. In competitive markets selection of buyers lead to lead to no differences in sale price among seller groups. The discrimination in the share of feedback (and time to feedback) might have implication in less competitive markets on eBay since the same feedback is used across all markets on the platform.

### Limitations and robustness

In this section, we briefly discuss the limitations to our findings. First, we collected information manually; this may give rise to measurement errors. We expect these potential errors to be stable across seller groups and therefore not affect our results.

Second, buyers could select sellers based on whether the username is anonymous or not. Looking at each of the four seller name groups, the share of female buyers and foreign buyers are similar across anonymous and non-anonymous sellers alike (see Table I and J in S1 Appendix). On observable characteristics, such as gender and foreignness, there are no differences in buyers by the username of the sellers.

Last, we have some information about the voucher market before and after we ran the experiment. We collected a data set in August 2011, one year before the experiment and September 2012, the month following the end of the experiment (see Table K in S1 Appendix). The average price is around 9 SEK higher than the average price of our sellers. The sellers in the 2011 data set are most likely more experienced than the sellers in our experiment. This price difference is in line with the economic returns to the feedback we find in the paper. In Table K in S1 Appendix, we also display the share of anonymous sellers on these two occasions. The share of anonymous sellers in August 2011 and September 2012 is 40%. In sum, anonymous usernames among sellers are common, and the experiment did not seem to directly affect the sale price in the market.

*Sample size and multiple testing*. At the time of the experiment, we were not aware of the now well-debated advantages of pre-registration of experimental studies. We considered the sample of 400 auctions large enough. Using Equation 6 in [62], assuming an equal size of the comparison groups, a power of 80% and a significance level of 0.05 we have a minimum detectable effect size of 0.5 standard deviations in the sale price. This effect size is, for example, higher compared to [27]. It can be useful to calculate a post-hoc power for studies with small sample sizes [63]. The paper shows that to explore the probability of the estimate having the wrong sign and being exaggerated if the sample is small. Assuming an unbiased estimate, problems with exaggeration arise when power is below 0.5, and problems with incorrect sign arise when power is below 0.1. We conducted a rough post-hoc power calculation on the two significant results in regression 2 in Table 6, pertaining to the anonymous setting. Here we combine the coefficient from the interaction and the coefficient from the non-anonymous setting for the respective group comparisons. Using the R code by [63] include the standard error from the above-mentioned regressions and the effect sizes from [27] of around 0.2, our study seems to have had enough power that it is unproblematic to disregard potential issues of exaggeration or wrong sign.

We did not take multiple testing into account when deciding on the number of observations for the experiment, and conducting multiple tests can increase the probability of Type I errors. To avoid an increase of Type II error, we did not include corrections for multiple testing in the main text. We primarily focus on 4 regressions with 3 coefficient in each. With 12 test coefficients and a size of 0.05, the probability of at least one rejection is 0.460. A Bonferroni adjustment of the nominal size to 0.005/12 = 0.004 provide us with an actual size (true significance) of 0.05. Applying this to our results, the interaction effects are not significant, but we still find indications that anonymous foreign male sellers receive less feedback than non-foreign female sellers. The conclusion of the study stands—in a market with many buyers and sellers discriminatory buyers to select sellers, but cannot influence the market outcome (sale price). Before drawing further inferences, however, there is a need for replications across contexts.

*Sample restrictions*. In the analysis, 20 observations where the buyer interacted with the seller in a previous auction were included in the sample. To exclude potential dependencies between observations, we restricted the sample to include the first auction where a specific buyer and seller interacted. Also, we deleted 11 observations where we received more than one email from the buyer. In total, this amounts to a sample of 405 observations. We then ran the regressions using the provision of feedback as the dependent variable. The sample provides us with the same results (see Table L in S1 Appendix). Our results do not seem to depend on the specificity of the main sample.

### Discussion

This study shows that, in a competitive market, buyers with an animus cannot directly influence the market outcome. In the experiment, we keep half the names of the sellers anonymous during the bidding process and the other half visible, and we show that buyers select sellers on names when information is available. We find discrimination in the feedback rating when the buyer could not see the seller name during the bidding process. This is likely because discriminatory bidders select out of the auctions of sellers with foreign-sounding names, leaving winners who do not discriminate. We could expect auctions of sellers with foreign-sounding names to receive fewer bidders and lower auction prices. However, we observe no differences in the sale price or the number of bids received by sellers with foreign or domestic sounding names.

The seller’s social identity matter for building a reputation online. We have too little data to probe into how the buyer identity matter for discrimination in the feedback ratings. Fewer feedback ratings on eBay can influence other eBay markets and may have implications for future feedback, future sales, and prices, in particular on less competitive markets. Another implication of our study concerns anonymity as a means to avoid discrimination and get fair treatment. Our experiment shows that this might backfire if the platform provides identifying information later in the process.

Our experimental design can investigate the direct effects of selection on discrimination in market outcomes. The economic argument that competition eradicates discrimination is a preference selection effect. When sellers are anonymous, there can be no buyer selection into sellers’ auctions. The seller names then cause discrimination in buyer feedback. We can somewhat differentiate whether discrimination is taste-based or statistical discrimination. If some buyers had a strong dislike for sellers of particular groups, following [11], they could have decided not to pay the winning price once the name of the seller was revealed, i.e. bearing the cost of canceling the trade to avoid further interaction with the seller, like in [16]. However, none of the buyers in our study cancelled the trade, leading us to conclude that the discrimination we find is subtle. At the feedback stage, the buyers can form expectations of the sellers’ likelihood of returning the feedback, and this might depend on the social identity of the seller. However, when we as sellers provided the feedback first, only one-third of the buyers that received feedback from the seller first actually provided feedback to the seller in return. This result suggests that the results are not driven by statistical discrimination. The results thus lend support to a more implicit form of taste-based discrimination, see discussion in [64].

## Conclusion

The study highlights that in competitive markets with many buyers and sellers, discriminatory buyers will not be able to in a direct way, influence the market outcome. Opportunities to be anonymous online in economic exchange block the possibility to choose seller by name and individuals with discriminatory attitudes can end up buying products from sellers of their dislike. Our results open up avenues for future research to investigate how anonymity change selection on social cues in other economic settings.

## Supporting information

S1 Appendix Additional description on method and estimations. (PDF)

## References

[1] Anderson L, Fryer R, Holt C. Discrimination: experimental evidence from psychology and economics. Handbook on the Economics of Discrimination. 2006; p. 97–118.

[2] Pager D, Shepherd H. The sociology of discrimination: Racial discrimination in employment, housing, credit, and consumer markets. Annual review of sociology. 2008;34:181 10.1146/annurev.soc.33.040406.131740 20689680 PMC2915460

[3] Nunley JM, Owens MF, Howard RS. The effects of information and competition on racial discrimination: Evidence from a field experiment. Journal of Economic Behavior & Organization. 2011;80(3):670–679. 10.1016/j.jebo.2011.06.028

[4] Przepiorka W. Buyers pay for and sellers invest in a good reputation: More evidence from eBay. The Journal of Socio-Economics. 2013;42:31–42. 10.1016/j.socec.2012.11.004

[5] Ayres I, Banaji M, Jolls C. Race effects on eBay. The RAND Journal of Economics. 2015;46(4):891–917. 10.1111/1756-2171.12115

[6] Doleac JL, Stein LC. The visible hand: Race and online market outcomes. The Economic Journal. 2013;123(572):F469–F492. 10.1111/ecoj.12082

[7] Peitz M, Waldfogel J. The Oxford handbook of the digital economy. Oxford University Press; 2012.

[8] Cabral L, Hortacsu A. The dynamics of seller reputation: Evidence from eBay. The Journal of Industrial Economics. 2010;58(1):54–78. 10.1111/j.1467-6451.2010.00405.x

[9] Keser C. Experimental games for the design of reputation management systems. IBM Systems Journal. 2003;42(3):498–506. 10.1147/sj.423.0498

[10] Shapiro C. Consumer information, product quality, and seller reputation. The Bell Journal of Economics. 1982; p. 20–35. 10.2307/3003427

[11] Becker G. The economics of discrimination; 1957.

[12] Allport GW. The nature of prejudice. 1954.

[13] Phelps ES. The statistical theory of racism and sexism. The american economic review. 1972;62(4):659–661.

[14] Arrow K. The Theory of discrimination. in 0 Ashenfelter and Rees A. (Eds.) Discrimination in Labor Markets (pp. 3–33); 1973.

[15] Akerlof GA. Discriminatory, status-based wages among tradition-oriented, stochastically trading coconut producers. Journal of Political Economy. 1985;93(2):265–276. 10.1086/261299

[16] Hedegaard MS, Tyran JR. The Price of Prejudice. American Economic Journal: Applied Economics. 2018;10(1):40–63.

[17] Dellarocas C. The digitization of word of mouth: Promise and challenges of online feedback mechanisms. Management science. 2003;49(10):1407–1424. 10.1287/mnsc.49.10.1407.17308

[18] Bolton GE, Katok E, Ockenfels A. How effective are electronic reputation mechanisms? An experimental investigation. Management science. 2004;50(11):1587–1602. 10.1287/mnsc.1030.0199

[19] Lucking-Reiley D, Bryan D, Prasad N, Reeves D. Pennies from ebay: The determinants of price in online auctions*. The Journal of Industrial Economics. 2007;55(2):223–233. 10.1111/j.1467-6451.2007.00309.x

[20] Resnick P, Zeckhauser R, Swanson J, Lockwood K. The value of reputation on eBay: A controlled experiment. Experimental economics. 2006;9(2):79–101. 10.1007/s10683-006-4309-2

[21] Houser D, Wooders J. Reputation in auctions: Theory, and evidence from eBay. Journal of Economics & Management Strategy. 2006;15(2):353–369. 10.1111/j.1530-9134.2006.00103.x

[22] Livingston JA. How valuable is a good reputation? A sample selection model of internet auctions. Review of Economics and Statistics. 2005;87(3):453–465. 10.1162/0034653054638391

[23] Melnik MI, Alm J. Does a seller’s ecommerce reputation matter? Evidence from eBay auctions. The journal of industrial economics. 2002;50(3):337–349. 10.1111/1467-6451.00180

[24] Bertrand M, Mullainathan S. Are Emily and Greg more employable than Lakisha and Jamal? A field experiment on labor market discrimination. The American Economic Review. 2004;94(4):991–1013. 10.1257/0002828042002561

[25] Riach PA, Rich J. Field experiments of discrimination in the market place*. The economic journal. 2002;112(483):F480–F518. 10.1111/1468-0297.00080

[26] Lieberson S. A matter of taste: How names, fashions, and culture change. Yale University Press; 2000.

[27] Arai M, Bursell M, Nekby L. The Reverse Gender Gap in Ethnic Discrimination: Employer Stereotypes of Men and Women with Arabic Names. International Migration Review. 2015.

[28] Bolton G, Greiner B, Ockenfels A. Engineering trust: reciprocity in the production of reputation information. Management science. 2013;59(2):265–285. 10.1287/mnsc.1120.1609

[29] Ostrom E, Walker J. Trust and Reciprocity: Interdisciplinary Lessons for Experimental Research: Interdisciplinary Lessons for Experimental Research. Russell Sage Foundation; 2003.

[30] Lumeau M, Masclet D, Pénard T. Reputation and social (dis) approval in feedback mechanisms: An experimental study. Journal of Economic Behavior & Organization. 2015;112:127–140. 10.1016/j.jebo.2015.02.002

[31] Klein TJ, Lambertz C, Spagnolo G, Stahl KO. The actual structure of eBay’s feedback mechanism and early evidence on the effects of recent changes. SFB/TR 15 Discussion Paper; 2007.

[32] Jian L, MacKie-Mason JK, Resnick P. I scratched yours: The prevalence of reciprocation in feedback provision on eBay. The BE Journal of Economic Analysis & Policy. 2010;10(1).

[33] Dellarocas C, Wood CA. The sound of silence in online feedback: Estimating trading risks in the presence of reporting bias. Management Science. 2008;54(3):460–476. 10.1287/mnsc.1070.0747

[34] Greiner B, Levati MV. Indirect reciprocity in cyclical networks: An experimental study. Journal of Economic Psychology. 2005;26(5):711–731. 10.1016/j.joep.2004.04.003

[35] Cabral L. Reputation on the Internet. The Oxford Handbook of the Digital Economy. 2012; p. 343–354.

[36] Diekmann A, Jann B, Przepiorka W, Wehrli S. Reputation formation and the evolution of cooperation in anonymous online markets. American sociological review. 2014;79(1):65–85. 10.1177/0003122413512316

[37] Mauss M. The gift: The form and reason for exchange in archaic societies. Routledge; 2002.

[38] Nishimura N, Cason TN, Saijo T, Ikeda Y. Spite and reciprocity in auctions. Games. 2011;2(3):365–411. 10.3390/g2030365

[39] Fershtman C, Gneezy U. Discrimination in a segmented society: An experimental approach. Quarterly Journal of Economics. 2001; p. 351–377. 10.1162/003355301556338

[40] Buchan NR, Croson RT, Dawes RM. Swift Neighbors and Persistent Strangers: A Cross-Cultural Investigation of Trust and Reciprocity in Social Exchange1. American Journal of Sociology. 2002;108(1):168–206. 10.1086/344546

[41] Johansson-Stenman O, Mahmud M, Martinsson P. Trust and religion: Experimental evidence from rural Bangladesh. Economica. 2009;76(303):462–485. 10.1111/j.1468-0335.2008.00689.x

[42] Slonim R, Guillen P. Gender selection discrimination: Evidence from a trust game. Journal of Economic Behavior & Organization. 2010;76(2):385–405. 10.1016/j.jebo.2010.06.016

[43] Bouckaert J, Dhaene G. Inter-ethnic trust and reciprocity: results of an experiment with small businessmen. European Journal of Political Economy. 2004;20(4):869–886. 10.1016/j.ejpoleco.2003.08.006

[44] Glaeser EL, Laibson DI, Scheinkman JA, Soutter CL. Measuring trust. Quarterly Journal of Economics. 2000; p. 811–846. 10.1162/003355300554926

[45] Lessig L. Code 2.0: Code and Other Laws of Cyberspace; 2006.

[46] Eklund L. Doing gender in cyberspace: The performance of gender by female World of Warcraft players. Convergence. 2011;17(3):323–342. 10.1177/1354856511406472

[47] Krause A, Rinne U, Zimmermann KF. Anonymous job applications in Europe. IZA Journal of European Labor Studies. 2012;1(1):1 10.1186/2193-9012-1-5

[48] Goldin C, Rouse C. Orchestrating Impartiality: The Impact of “Blind” Auditions on Female Musicians. The american economic review. 2000;90(4):715–741. 10.1257/aer.90.4.715

[49] Edin PA, Lagerström J, et al. Blind dates: quasi-experimental evidence on discrimination. Institutet för arbetsmarknadspolitisk utvärdering (IFAU); 2006.

[50] Åslund O, Skans ON. Do anonymous job application procedures level the playing field? Industrial & labor relations review. 2012;65(1):82–107. 10.1177/001979391206500105

[51] Bøg M, Kranendonk E. Blindfolded Recruiting. Working paper, EEA-ESEM, February 15; 2013.

[52] Elfenbein, D. W., Fisman, R. J., McManus, B. The impact of socioeconomic and cultural differences on online trade SSRN, Working paper. 2018.

[53] Roth AE, Ockenfels A. Last-minute bidding and the rules for ending second-price auctions: Evidence from eBay and Amazon auctions on the Internet. American economic review. 2002;92(4):1093–1103. 10.1257/00028280260344632

[54] Zeithammer R, Adams C. The sealed-bid abstraction in online auctions. Marketing Science. 2010;29(6):964–987. 10.1287/mksc.1100.0561

[55] Anwar S, McMillan R, Zheng M. Bidding behavior in competing auctions: Evidence from eBay. European Economic Review. 2006;50(2):307–322. 10.1016/j.euroecorev.2004.10.007

[56] Peters M, Severinov S. Internet auctions with many traders. Journal of Economic Theory. 2006;130(1):220–245. 10.1016/j.jet.2005.04.005

[57] Cabral L, Li L. A Dollar for Your Thoughts: Feedback-Conditional Rebates on eBay. Management Science. 2015;61(9):2052–2063. 10.1287/mnsc.2014.2074

[58] Nakamura L. Digitizing race: Visual cultures of the Internet. vol. 23 U of Minnesota Press; 2008.

[59] Young A. Channeling fisher: Randomization tests and the statistical insignificance of seemingly significant experimental results. London School of Economics, Working Paper, Feb. 2016.

[60] Muchnik L, Aral S, Taylor SJ. Social influence bias: A randomized experiment. Science. 2013;341(6146):647–651. 10.1126/science.1240466 23929980

[61] Frey V, Van De Rijt A. Arbitrary inequality in reputation systems. Scientific reports. 2016;6:38304 10.1038/srep38304 27995957 PMC5171920

[62] List JA, Sadoff S, Wagner M. So you want to run an experiment, now what? Some simple rules of thumb for optimal experimental design. Experimental Economics. 2011;14(4):439–457. 10.1007/s10683-011-9275-7

[63] Gelman A, Carlin J. Beyond power calculations assessing type S (sign) and type M (magnitude) errors. Perspectives on Psychological Science. 2014;9(6):641–651. 10.1177/1745691614551642 26186114

[64] Fiske ST. What we know now about bias and intergroup conflict, the problem of the century. Current Directions in Psychological Science. 2002;11(4):123–128. 10.1111/1467-8721.00183

