# Supplementary material for: The effect of competition on discrimination in online markets—Anonymity and selection
Source: PLoS One. 2019 Aug 28;14(8):e0221857. doi: 10.1371/journal.pone.0221857 (PMC6713341; doi:10.1371/journal.pone.0221857)
Supplement: S1 Appendix — (PDF) [file pone.0221857.s001.pdf]

S1 - Appendix. Additional description on method and estimations.

Procedure Description of selection of seller names

The group of non-foreign sounding seller names is a convenience sample of individuals with common first names in Sweden (by the frequency at Statistics Sweden). We asked a convenience sample of twenty students at Stockholm University to match names of potential sellers to a gender (man or woman) and a region (coded according to Statistics Sweden: Latin America and South America, the Middle East and North Africa, EU15+, the rest of Europe, the rest of Asia, and the rest of Africa). We then asked the eight individuals where all students correctly matched gender and to the region of the Middle East and North Africa to collaborate with us. A similar process of categorizing names has been used by [1] and [2]. They found a high positive correlation between a judgment-based regional coding of names and the actual regions of birth. This result indicates that identifying the region of birth by observing an individual's name is relatively straightforward.

Table A. Logit marginal effects: Differences in share of feedback between male-foreign seller and other groups. Buyer characteristics included.

Name	Surname	Username
Daniel	Johansson	daniel012
Tomas	Larsson	tomas012
Savas	Calsikan	savasc
Alireza	Behtuoi	alirezaB
Pernilla	Andersson	pernilla04
Tone	Johansson	tone012
Khadra	Seerar	khadra01
Afamia	Maraha	afamia
Karl-Göran	Karlsson	kgka
Fredrik	Mattsson	frmat
Hossein	Ali	hoal01
Tiglat	Maraha	tima01
Louise	Johanesson	lojo01
Eva	Karlsson	evka0
Fatima	Nekshbandi	fane01
Doaa	Mohamed	domo01

Table B. Logit marginal effects: Differences in share of feedback between male-foreign seller and other groups. Buyer characteristics included.

	(1) Non-anonymous	(2) Non-anonymous	(3) Anonymous	(4) Anonymous
Male non-foreign seller	-0.069	-0.082	0.106	0.143*
	(0.094)	(0.087)	(0.070)	(0.062)
Female non-foreign seller	-0.050	-0.007	0.163*	0.178**
	(0.095)	(0.082)	(0.068)	(0.061)
Female foreign seller	-0.040	-0.050	0.073	0.046
	(0.089)	(0.083)	(0.070)	(0.070)
Price		0.003		-0.005
		(0.004)		(0.003)
Female buyer		-0.206**		-0.086
		(0.064)		(0.066)
Foreign buyer		0.147*		-0.185*
		(0.068)		(0.076)
Big city		-0.069		-0.051
		(0.086)		(0.085)
Buyer feedback		0.001*		0.000
		(0.000)		(0.000)
Buyer negative feedback		-0.152		-0.105
		(0.081)		(0.082)
New buyer		-0.156*		-0.244**
		(0.077)		(0.083)
New seller		-0.083		-0.116
		(0.077)		(0.068)
Number of bids		0.002		0.002
		(0.002)		(0.002)
Start-day	No	Yes	No	Yes
Observations	218	218	218	218

Standard errors in parentheses are clustered on buyer username.

* $p < 0.05$, ** $p < 0.01$, *** $p < 0.001$

Table C. OLS: Discrimination in sale price between male-foreign seller and other groups. Buyer characteristics included.

	(1)<br>Non-anonymous	(2)<br>Non-anonymous	(3)<br>Anonymous	(4)<br>Anonymous
Male non-foreign seller	2.113	2.759	2.047	0.869
	(2.081)	(1.607)	(1.908)	(1.755)
Female non-foreign seller	0.950	0.386	1.273	-0.679
	(2.048)	(1.656)	(2.130)	(1.685)
Female foreign seller	1.270	0.800	-0.583	-1.223
	(1.983)	(1.557)	(1.847)	(1.581)
Big city		1.276		1.549
		(1.658)		(1.522)
Female buyer		-0.757		-0.921
		(1.364)		(1.409)
Non-foreign buyer		0.875		2.793
		(1.534)		(1.552)
Buyer feedback		-0.002		-0.003*
		(0.002)		(0.001)
Buyer negative feedback		1.550		1.777
		(1.671)		(1.415)
New buyer		0.710		1.659
		(1.491)		(1.648)
New seller		-13.707***		-10.999***
		(1.368)		(1.226)
Number of bids		-0.019		0.013
		(0.040)		(0.044)
Constant	138.333***	147.095***	139.527***	144.677***
	(1.562)	(2.522)	(1.424)	(2.702)
Start-day	No	Yes	No	Yes
Adjusted R^2	-0.009	0.350	-0.004	0.256
Observations	218	218	218	218

Standard errors in parentheses are clustered on buyer username.

* $p < 0.05$, ** $p < 0.01$, *** $p < 0.001$

Table D. OLS: Sale price discrimination. Interaction of new seller and seller identity.

	(1) Non-anonymous new sellers
Male non-foreign seller	4.359
	(2.650)
Female non-foreign seller	-0.299
	(2.438)
Female foreign seller	1.461
	(2.139)
New seller	-13.764***
	(2.410)
New sellerXMale non-foreign seller	-2.229
	(3.398)
New sellerXFemale non-foreign seller	1.852
	(3.283)
New sellerXFemale foreign seller	-0.873
	(3.311)
Number of bids	-0.008
	(0.040)
Constant	147.447***
	(2.313)
Start-day and street name	Yes
Adjusted R^2	0.352
Observations	218

Standard errors in parentheses are clustered on buyer username.

* $p < 0.05$, ** $p < 0.01$, *** $p < 0.001$

Table E. Feedback discrimination using female foreign seller as reference group.

	(1) Non-anonymous	(2) Non-anonymous	(3) Anonymous	(4) Anonymous
Male non-foreign seller	-0.028	-0.031	0.036	0.103
	(0.087)	(0.080)	(0.079)	(0.072)
Female non-foreign seller	-0.009	0.041	0.098	0.140**
	(0.083)	(0.065)	(0.074)	(0.070)
Male foreign seller	0.039	0.048	-0.080	-0.048
	(0.083)	(0.077)	(0.084)	(0.078)
Controls	No	Yes	No	Yes
Observations	218	218	218	218

Standard errors in parentheses are clustered on buyer username.

* $p < 0.10$, ** $p < 0.05$, *** $p < 0.01$

Table F. Feedback discrimination. Dropping buyers when seller provide feedback first.

	(1)<br>No controls	(2)<br>Controls
Male non-foreign seller	0.085	0.249*
	(0.084)	(0.108)
Female non-foreign seller	0.174*	0.152
	(0.080)	(0.085)
Female foreign seller	0.058	0.149
	(0.084)	(0.098)
Price		-0.005
		(0.004)
New seller		-0.142
		(0.083)
Number of bids		0.001
		(0.002)
Start-day and street name	No	Yes
Adjusted R^2		
Observations	185	185

Standard errors clustered on buyer username.

* $p < 0.10$, ** $p < 0.05$, *** $p < 0.01$

Table G. Feedback discrimination. New seller and buyer less than 5 feedback.

	(1) Non-anonymous	(2) Non-anonymous	(3) Anonymous	(4) Anonymous
Male non-foreign seller	-0.069	-0.075	0.106	0.147**
	(0.094)	(0.086)	(0.070)	(0.062)
Female non-foreign seller	-0.050	0.004	0.163**	0.178***
	(0.095)	(0.082)	(0.068)	(0.064)
Female foreign seller	-0.040	-0.047	0.073	0.046
	(0.089)	(0.083)	(0.070)	(0.072)
New buyer 5		-0.183*		-0.175**
		(0.094)		(0.087)
New seller 5		-0.074		-0.112
		(0.081)		(0.076)
Controls	No	Yes	No	Yes
Adjusted R^2				
Observations	218	218	218	218

Standard errors clustered on buyer username.

* $p < 0.10$, ** $p < 0.05$, *** $p < 0.01$

Table H. Observable characteristics by anonymous vs. non-anonymous seller username.

	(1)<br>Price	(2)<br>Feedback	(3)<br>Non-foreign	(4)<br>Female	(5)<br>Big City	(6)<br>New buyer	(7)<br>Negative feeback	(8)<br>Buyer feedback
anonymity	0.766	0.009	-0.014	-0.064	-0.014	0.037	-0.060	-26.555
	(1.015)	(0.042)	(0.046)	(0.048)	(0.039)	(0.041)	(0.045)	(36.970)
Constant	139.445***							171.408***
	(0.884)							(29.563)
Adjusted R^2	-0.001							-0.001
Observations	436	436	436	436	436	436	436	436

Standard errors are clustered on buyer username.

** $p < 0.05$, *** $p < 0.01$, *** $p < 0.001$

Table I. Share of female buyers within seller name group by anonymity.

	(1) MFS	(2) FFS	(3) MNFS	(4) FNFS
anonymity	-0.079	0.024	-0.183*	-0.016
	(0.097)	(0.094)	(0.094)	(0.096)
Constant	0.588***	0.569***	0.554***	0.434***
	(0.070)	(0.065)	(0.066)	(0.069)
Adjusted R^2	-0.003	-0.009	0.025	-0.009
Observations	106	112	110	108

The abbrevations relate to the seller name categories.

* $p < 0.10$, ** $p < 0.05$, *** $p < 0.01$

Table J. Share of foreign buyers within seller name group by anonymity.

	(1) MFS	(2) FFS	(3) MNFS	(4) FNFS
anonymity	-0.005	-0.116	0.067	0.118
	(0.091)	(0.092)	(0.091)	(0.089)
Constant	0.314***	0.431***	0.304***	0.245***
	(0.065)	(0.064)	(0.064)	(0.063)
Adjusted R^2	-0.010	0.005	-0.004	0.007
Observations	106	112	110	108

The abbrevations relate to the seller name categories.

* $p < 0.10$, ** $p < 0.05$, *** $p < 0.01$

Table K. Summary of the market in 2011, during the experiment and September 2012

	(1)		
	n	mean	sd
Price Aug. 2011	31	147.807	22.502
Price	436	139.828	10.674
Price Sept. 2012	65	148.585	8.757
Number of bids Aug. 2011	31	9.355	8.781
Number of bids in experiment	436	27.683	14.625
Number of bids Sept. 2012	65	39.046	21.336
Share of anonymous sellers Aug. 2011	31	0.584	0.501
Share of anonymous sellers experiment	436	0.500	0.501
Share of anonymous sellers Sept. 2012	65	0.569	0.499

Table L. Feedback discrimination. Restricted sample.

	(1) Non-anonymous	(2) Non-anonymous	(3) Anonymous	(4) Anonymous
Male non-foreign seller	-0.087	-0.109	0.102	0.131*
	(0.096)	(0.089)	(0.075)	(0.068)
Female non-foreign seller	-0.049	-0.019	0.146**	0.162**
	(0.097)	(0.087)	(0.073)	(0.067)
Female foreign seller	-0.030	-0.052	0.053	0.028
	(0.092)	(0.087)	(0.078)	(0.078)
Controls	No	Yes	No	Yes
Adjusted R^2				
Observations	206	206	199	199

Standard errors clustered on buyer username.

* $p < 0.10$, ** $p < 0.05$, *** $p < 0.01$

Table M. Young: Logit marginal effects: - Differences in feedback between anonymous male-foreign seller and other groups

	rand.-c			rand.-t			
	**min.**	**max.**	**rand.**	**min.**	**max.**	**rand.**	**successful**
**Seller groups**	**p-value**	**p-value**	**p-value**	**p-value**	**p-value**	**p-value**	**iterations**
Male non-foreign	0.06494	0.06593	0.06573	0.04995	0.05095	0.05075	1000
Female non-foreign	0.01399	0.01499	0.01414	0.01099	0.01199	0.01115	1000
Female foreign	0.50849	0.50949	0.50887	0.48851	0.48951	0.48889	1000
Joint test	0.06394	0.06494	0.06446	0.05295	0.05395	0.05348	1000

Randomization test by Young (forthcoming).

Table N. Young: Logit - Differences in feedback between non-anonymous male-foreign seller and other groups

	rand.-c			rand.-t			
	**min.**	**max.**	**rand.**	**min.**	**max.**	**rand.**	**successful**
**Seller groups**	**p-value**	**p-value**	**p-value**	**p-value**	**p-value**	**p-value**	**iterations**
Male non-foreign	0.38262	0.38362	0.38342	0.40559	0.40659	0.40639	1000
Female non-foreign	0.88012	0.88112	0.88028	0.87912	0.88012	0.87928	1000
Female foreign	0.52547	0.52647	0.52585	0.53047	0.53147	0.53084	1000
Joint test	0.78422	0.78521	0.78474	0.79421	0.7952	0.79473	1000

Randomization test by Young (forthcoming).

Table O. Young: OLS - Differences in sale price between anonymous male-foreign seller and other groups

	rand.-c			rand.-t			
	min.	max.	rand.	min.	max.	rand.	successful
Seller groups	p-value	p-value	p-value	p-value	p-value	p-value	iterations
Male non-foreign	0.66733	0.66833	0.66813	0.66234	0.66334	0.66314	1000
Female non-foreign	0.7003	0.7013	0.70046	0.68531	0.68631	0.68547	1000
Female foreign	0.48651	0.48751	0.48689	0.44955	0.45055	0.44992	1000
Joint test	0.67033	0.67133	0.67086	0.63836	0.63936	0.63889	1000

Randomization test by Young (forthcoming).

Table P. Young: OLS - Differences in sale price between non-anonymous male-foreign seller and other groups

	rand.-c			rand.-t			
	**min.**	**max.**	**rand.**	**min.**	**max.**	**rand.**	**successful**
**Seller groups**	**p-value**	**p-value**	**p-value**	**p-value**	**p-value**	**p-value**	**iterations**
Male non-foreign	0.1029	0.1039	0.1037	0.07792	0.07892	0.07872	1000
Female non-foreign	0.80719	0.80819	0.80735	0.7952	0.7962	0.79536	1000
Female foreign	0.65634	0.65734	0.65672	0.61239	0.61339	0.61276	1000
Joint test	0.35964	0.36064	0.36017	0.35265	0.35365	0.35318	1000

Randomization test by Young (forthcoming).

Table Q. OLS: Discrimination in time to feedback.

	(1) Non-anonymous	(2) Non-anonymous	(3) Anonymous	(4) Anonymous
Male non-foreign seller	0.237	0.868	4.849*	5.020*
	(1.784)	(1.895)	(2.415)	(2.336)
Female non-foreign seller	2.410	2.021	3.460	4.488
	(2.242)	(2.405)	(2.197)	(2.427)
Female foreign seller	0.230	0.495	4.034*	4.942*
	(1.415)	(1.674)	(2.026)	(2.132)
Constant	7.184***	12.869	6.176***	27.053
	(1.123)	(8.851)	(0.837)	(19.729)
Controls	No	Yes	No	Yes
Adjusted R^2	-0.006	0.008	0.005	0.069

Standard errors in parentheses are clustered on buyer username.

* $p < 0.05$, ** $p < 0.01$, *** $p < 0.001$

Table R. No. unique bidders across situation and seller groups.

	(1)<br>Anonymity vs Non Anonymity	(2)<br>Non-Anonymity	(3)<br>Anonymity
Anonymity	-0.137		
	(0.235)		
Male non-foreign seller		0.208	0.576
		(0.460)	(0.465)
Female foreign seller		0.430	-0.202
		(0.460)	(0.468)
Female non-foreign seller		0.271	0.514
		(0.466)	(0.489)
Constant	7.887***	7.659***	7.543***
	(0.163)	(0.327)	(0.321)
Adjusted R^2	-0.002	-0.012	0.005
Observations	341	177	164

Standard errors in parentheses

* $p < 0.10$, ** $p < 0.05$, *** $p < 0.01$

References

1. Arai M, Bursell M, Nekby L. The Reverse Gender Gap in Ethnic Discrimination: Employer Stereotypes of Men and Women with Arabic Names. International Migration Review. 2015;.

2. Arai M, Thoursie PS. Renouncing personal names: An empirical examination of surname change and earnings. Journal of Labor Economics. 2009;27(1):127–147.
